# Cancer incidence in the south Asian population of England (1990–92)

**DOI:** 10.1038/sj.bjc.6690102

**Published:** 1999-02

**Authors:** H Winter, K K Cheng, C Cummins, R Maric, P Silcocks, C Varghese

**Affiliations:** Department of Public Health and Epidemiology, The University of Birmingham, Edgbaston, Birmingham, B15 2TT, UK; Trent Cancer Registry, Floor 6 Whitham Road, Sheffield, S10 2SJ, UK; Yorkshire Cancer Registry, Centre for Cancer Research, University of Leeds, Cookridge Hospital, Leeds, LS16 6QB, UK

**Keywords:** cancer incidence, south Asian, migrants, England

## Abstract

Cancer incidence among English south Asians (residents in England with ethnic origins in India, Pakistan or Bangladesh) is described and compared with non-south Asian and Indian subcontinent rates. The setting for the study was areas covered by Thames, Trent, West Midlands and Yorkshire cancer registries. The study identified 356 555 cases of incident cancer (ICD9:140–208) registered between 1990 and 1992, including 3845 classified as English south Asian. The main outcome measures were age specific and directly standardized incidence rates for all cancer sites (ICD9:140–208). English south Asian incidence rates for all sites combined were significantly lower than non-south Asian rates but higher than Indian subcontinent rates. English south Asian rates were substantially higher than Indian subcontinent rates for a number of common sites including lung cancer in males, breast cancer in females and lymphoma in both sexes. English south Asian rates for childhood and early adult cancer (0–29 years) were similar or higher than non-south Asian rates. English south Asian rates were significantly higher than non-south Asian rates for Hodgkin's disease in males, cancer of the tongue, mouth, oesophagus, thyroid gland and myeloid leukaemia in females, and cancer of the hypopharynx, liver and gall bladder in both sexes. The results are consistent with a transition from the lower cancer risk of the country of ethnic origin to that of the country of residence. They suggest that detrimental changes in lifestyle and other exposures have occurred in the migrant south Asian population. © 1999 Cancer Research Campaign


*British Journal of Cancer* (1999) **79**(3/4), 645–654
© 1999 Cancer Research Campaign
Article no. bjoc.1998.0102

### Cancer incidence in the south Asian population of England (1990–92)

**H Winter[1], KK Cheng[1], C Cummins[1], R Maric[1], P Silcocks[2] and C Varghese[3]**

[1]Department of Public Health and Epidemiology, The University of Birmingham, Edgbaston, Birmingham B15 2TT, UK; [2]Trent Cancer Registry, Floor 6, Weston Park Hospital NHS Trust, Whitham Road, Sheffield S10 2SJ, UK; [3]Yorkshire Cancer Registry, Centre for Cancer Research, University of Leeds, Arthington House, Cookridge Hospital, Leeds LS16 6QB, UK

**Summary** Cancer incidence among English south Asians (residents in England with ethnic origins in India, Pakistan or Bangladesh) is described and compared with non-south Asian and Indian subcontinent rates. The setting for the study was areas covered by Thames, Trent, West Midlands and Yorkshire cancer registries. The study identified 356 555 cases of incident cancer (ICD9:140–208) registered between 1990 and 1992, including 3845 classified as English south Asian. The main outcome measures were age specific and directly standardized incidence rates for all cancer sites (ICD9:140–208). English south Asian incidence rates for all sites combined were significantly lower than non-south Asian rates but higher than Indian subcontinent rates. English south Asian rates were substantially higher than Indian subcontinent rates for a number of common sites including lung cancer in males, breast cancer in females and lymphoma in both sexes. English south Asian rates for childhood and early adult cancer (0–29 years) were similar or higher than non-south Asian rates. English south Asian rates were significantly higher than non-south Asian rates for Hodgkin's disease in males, cancer of the tongue, mouth, oesophagus, thyroid gland and myeloid leukaemia in females, and cancer of the hypopharynx, liver and gall bladder in both sexes. The results are consistent with a transition from the lower cancer risk of the country of ethnic origin to that of the country of residence. They suggest that detrimental changes in lifestyle and other exposures have occurred in the migrant south Asian population.

**Keywords:** cancer incidence; south Asian; migrants; England

Approximately 3% of the population of England have their origins in the Indian subcontinent. To date, only proportional mortality and standardized mortality ratios based on country of origin have been described (Balarajan et al, 1984; Swerdlow et al, 1995; Balarajan et al, 1997), and there has been no comprehensive study of cancer incidence in this population. Published studies on incidence are limited to relatively small numbers because they were based on specific geographical areas (Donaldson et al, 1984; Matheson et al, 1985; Barker et al, 1990) or on children alone (Stiller et al, 1991; Muir et al, 1992; Powell et al, 1994; Varghese et al, 1996). One non-UK study has examined cancer incidence in migrants to Australia from a number of Asian countries including India and Sri Lanka (Grulich et al, 1995). For the first time, the 1991 census provided population figures based on ethnic origin (OPCS, 1993*a*). In this study, we combined these denominator figures with numerator data from cancer registrations in the years 1990–92 to study the incidence rates of cancer among south Asians in four regional cancer registries in England.

As the study population incorporates approximately 80% of the south Asian population resident in England, our estimates on incidence should be more precise than those available hitherto. The results constitute an important baseline for monitoring trends and health care needs assessment in these ethnic groups. Comparison of standardized rates with those for the Indian subcontinent and the non-south Asian study population would also serve to indicate how rates in south Asians have changed with migration and should provide data for the generation of aetiological hypotheses.

*Revised 26 May 1998*
*Received 27 January 1998*
*Accepted 15 June 1998*

*Correspondence to:* C Cummins, Department of Public Health and Epidemiology, The University of Birmingham, Edgbaston, Birmingham B15 2TT, UK

### PATIENTS AND METHODS

For the purpose of this study, a south Asian was defined as a person, irrespective of birthplace, with an ethnic origin in peoples indigenous to India, Pakistan or Bangladesh.

The study population consisted of those persons resident in areas covered by Thames, Trent, West Midlands and Yorkshire cancer registries, representing approximately 57% of the total population of England. As cancer registry data on ethnic origin were largely incomplete, cancer cases were classified as south Asian if their names were identified as having an Indian, Pakistani or Bangladeshi, but not Sri Lankan, origin.

The full list of cancer registrations for the four regions (1990–92) was run against the Nam Pehchan computer software package (Bradford Health Authority) (subsequently referred to as Nam Pehchan), developed by Bradford Health Authority and the City of Bradford Metropolitan Council, to identify partial or complete matches against the program's dictionary of south Asian names. In a pilot study, Nam Pehchan achieved 91.0% sensitivity, 99% specificity and a positive predictive value of 87.5% when run against a West Midlands childhood cancer data set in which ethnicity had been identified by the West Midlands Regional Children's Tumour Research Group from hospital notes and consultations (440 south Asian cases out of a total of 6437). As the program's dictionary of names may not yet be fully comprehensive and as some names were classified as south Asian on the basis

**Table 1** Age-standardized incidence rates (males) per 100 000 person–years (1990–92)

| | | English registries | | | | | | Indian registry range[a] |
|---|---|---|---|---|---|---|---|---|
| | | South Asian | | | Non-south Asian | | | |
| ICD9 | Site | *n* | Rate | 95% CI | *n* | Rate | 95% CI | |
| 140 | Lip | 0 | | | 135 | 0.2 | 0.1–0.3 | 0.0–0.6 |
| 141 | Tongue | 23 | 1.7 | 0.9–2.5 | 635 | 1.1 | 1.0–1.3 | 3.4–14.0 |
| 142 | Salivary glands | 9 | 0.8 | 0.2–1.4 | 289 | 0.5 | 0.4–0.6 | 0.3–0.7 |
| 143–5 | Mouth | 26 | 1.9 | 1.0–2.7 | 838 | 1.5 | 1.4–1.7 | 4.4–6.9 |
| 146 | Oropharynx | 5 | 0.5 | 0.0–1.0 | 414 | 0.8 | 0.6–0.9 | 1.7–6.2 |
| 147 | Nasopharynx | 9 | 0.5 | 0.1–0.8 | 187 | 0.4 | 0.3–0.5 | 0.5–0.7 |
| 148 | Hypopharynx | 21 | 2.0 | 1.0–3.0 | 331 | 0.6 | 0.4–0.7 | 5.3–10.8 |
| 149 | Other mouth/pharynx | 5 | 0.4 | 0.0–0.9 | 169 | 0.3 | 0.2–0.4 | 0.6–2.2 |
| 150 | Oesophagus | 39 | 3.3 | 2.1–4.5 | 4878 | 7.7 | 7.5–8.0 | 7.6–11.4 |
| 151 | Stomach | 70 | 5.6 | 4.2–7.1 | 9622 | 14.4 | 14.1–14.8 | 2.1–15.1 |
| 152 | Small intestine | 4 | 0.3 | 0.0–0.6 | 325 | 0.5 | 0.4–0.6 | 0.2–0.3 |
| 153 | Colon | 88 | 7.4 | 5.7–9.1 | 12 191 | 18.6 | 18.2–19.0 | 1.5–3.2 |
| 154 | Rectum | 65 | 4.9 | 3.6–6.2 | 9038 | 14.2 | 13.9–14.6 | 2.4–4.6 |
| 155 | Liver | 85 | 6.2 | 4.7–7.7 | 1219 | 2.0 | 1.8–2.2 | 1.7–3.4 |
| 156 | Gall bladder | 26 | 2.2 | 1.2–3.2 | 845 | 1.3 | 1.1–1.4 | 0.4–1.0 |
| 157 | Pancreas | 47 | 4.4 | 3.0–5.8 | 4733 | 7.3 | 7.1–7.6 | 0.7–2.5 |
| 158 | Peritoneum | 9 | 0.5 | 0.1–0.9 | 185 | 0.3 | 0.2–0.4 | 0.3–0.7 |
| 160 | Nasal cavities | 6 | 0.5 | 0.0–0.9 | 341 | 0.6 | 0.5–0.7 | 0.6–1.0 |
| 161 | Larynx | 48 | 3.9 | 2.6–5.1 | 2386 | 4.0 | 3.8–4.2 | 4.3–10.2 |
| 162 | Trachea/bronchus/lung | 344 | 30.1 | 26.7–33.5 | 38 249 | 58.1 | 57.5–58.8 | 8.5–14.0 |
| 163 | Pleura | 10 | 0.8 | 0.2–1.3 | 1168 | 2.0 | 1.8–2.2 | 0.1–0.8 |
| 164 | Thymus/heart/mediastinum | 4 | 0.3 | 0.0–0.6 | 153 | 0.3 | 0.2–0.4 | 0.1–0.2 |
| 170 | Bone | 21 | 1.1 | 0.6–1.6 | 344 | 0.8 | 0.7–0.9 | 0.7–1.2 |
| 171 | Connective/soft tissue | 33 | 2.1 | 1.3–3.0 | 903 | 1.8 | 1.6–1.9 | 0.9–2.4 |
| 172 | Melanoma of skin | 7 | 0.6 | 0.1–1.1 | 2096 | 4.0 | 3.8–4.2 | 0.2–0.4 |
| 173 | Non-melanoma skin | 26 | 2.0 | 1.1–2.9 | 15 772 | 25.0 | 24.6–25.5 | 1.2–1.9 |
| 175 | Male breast | 3 | 0.2 | 0.0–0.6 | 321 | 0.5 | 0.4–0.6 | 0.2–0.5 |
| 185 | Prostate | 159 | 16.7 | 13.9–19.4 | 21 447 | 29.0 | 28.6–29.5 | 2.1–6.9 |
| 186 | Testis | 28 | 1.7 | 0.9–2.5 | 1990 | 4.4 | 4.2–4.7 | 0.6–1.0 |
| 187 | Penis, other male genital | 5 | 0.5 | 0.0–1.1 | 513 | 0.9 | 0.7–1.0 | 1.7–2.8 |
| 188 | Bladder | 102 | 8.4 | 6.6–10.2 | 12 614 | 19.2 | 18.8–19.6 | 1.8–3.6 |
| 189 | Kidney | 49 | 3.3 | 2.3–4.4 | 4032 | 6.9 | 6.6–7.2 | 1.1–1.4 |
| 190 | Eye | 9 | 0.6 | 0.1–1.0 | 323 | 0.7 | 0.5–0.8 | 0.1–0.6 |
| 191–2 | Brain/nervous system | 95 | 5.5 | 4.3–6.8 | 2981 | 6.2 | 5.9–6.5 | 1.3–2.4 |
| 193 | Thyroid gland | 13 | 0.8 | 0.3–1.3 | 333 | 0.6 | 0.5–0.7 | 0.5–0.9 |
| 194 | Other endocrine glands | 10 | 0.6 | 0.2–1.0 | 271 | 0.7 | 0.5–0.8 | 0.1–1.6 |
| 200–3 | Lymphoma | 246 | 16.8 | 14.5–19.1 | 8335 | 15.0 | 14.6–15.4 | 4.8–5.7 |
| 201 | Hodgkin's disease | 64 | 3.5 | 2.5–4.4 | 1028 | 2.4 | 2.1–2.5 | 1.2–1.6 |
| 200/2 | Non-Hodgkin's lymphoma | 143 | 9.9 | 8.1–11.7 | 5305 | 9.5 | 9.2–9.8 | 2.6–3.7 |
| 203 | Multiple myeloma | 39 | 3.4 | 2.2–4.6 | 2002 | 3.1 | 2.9–3.3 | 0.7–0.9 |
| 204–8 | Leukaemia | 106 | 6.9 | 5.4–8.4 | 4045 | 7.4 | 7.1–7.7 | 2.7–4.0 |
| 204 | Lymphoid leukaemia | 55 | 3.3 | 2.3–4.3 | 1851 | 3.7 | 3.5–4.0 | 1.0–1.6 |
| 205 | Myeloid leukaemia | 50 | 3.5 | 2.4–4.7 | 1951 | 3.3 | 3.1–3.5 | 1.1–1.8 |
| 206–8 | Other leukaemia | 1 | 0.1 | 0.0–0.3 | 243 | 0.4 | 0.3–0.5 | 0.3–0.7 |
| | Primary site uncertain[b] | 103 | 8.7 | 6.8–10.6 | 10 414 | 15.9 | 15.5–16.3 | 6.0–25.2 |
| | All sites, excluding 173 | 1932 | 152.8 | 145.3–160.2 | 159 293 | 250.9 | 249.5–252.2 | 93.9–140.7 |

*n* represents number of cases. [a]Indian rates are based on 1983–87 data. [b]Primary site uncertain includes the following ICD9 codes: 159, 165, 195–199.

of a partial stem match, the entire dataset was also screened using the strategy which follows.

A dictionary of common non-south Asian names was generated from the full list of names and used to exclude those program negatives in which all component names, i.e. surname/forename(s)/maiden name, matched against an entry in the non-south Asian dictionary. The full list of program positives and those program negatives not excluded by the above procedure was visually inspected and cases classified as non-south Asian in which all component names were independently identified as non-south Asian by three members of the research team. The full list of remaining names, excluding those classified as non-south Asian by one of the above procedures, was submitted to a panel for final classification. The panel was made up of three core members representing the main south Asian religious groups found in the United Kingdom (Hindu/Moslem/Sikh) and five additional contributors with knowledge of a range of south Asian and non-south Asian Moslem populations. Books, providing lists of Hindu/Moslem/Sikh names were available throughout, to both researchers and panel members (Patel, 1992;

 *© Cancer Research Campaign 1999*

**Table 2** Age-standardized incidence rates (females) per 100 000 person–years (1990–92)

| | | English registries | | | | | | Indian registry range[a] |
|---|---|---|---|---|---|---|---|---|
| | | South Asian | | | Non-south Asian | | | |
| ICD9 | Site | n | Rate | 95% CI | n | Rate | 95% CI | |
| 140 | Lip | 0 | | | 53 | 0.1 | 0.0–0.1 | 0.1–0.5 |
| 141 | Tongue | 25 | 1.9 | 1.1–2.8 | 407 | 0.5 | 0.4–0.6 | 1.1–2.5 |
| 142 | Salivary glands | 6 | 0.4 | 0.0–0.9 | 266 | 0.4 | 0.3–0.5 | 0.4–0.5 |
| 143–5 | Mouth | 29 | 2.8 | 1.7–3.9 | 509 | 0.7 | 0.6–0.8 | 3.7–9.6 |
| 146 | Oropharynx | 5 | 0.4 | 0.0–0.9 | 154 | 0.2 | 0.1–0.3 | 0.3–1.1 |
| 147 | Nasopharynx | 2 | 0.1 | 0.0–0.3 | 102 | 0.2 | 0.1–0.3 | 0.2–0.4 |
| 148 | Hypopharynx | 7 | 0.5 | 0.1–1.0 | 194 | 0.3 | 0.2–0.4 | 1.2–2.2 |
| 149 | Other mouth/pharynx | 2 | 0.2 | 0.0–0.5 | 86 | 0.1 | 0.0–0.2 | 0.3–0.7 |
| 150 | Oesophagus | 47 | 4.6 | 3.1–6.0 | 3342 | 3.3 | 3.2–3.5 | 5.6–8.8 |
| 151 | Stomach | 40 | 4.1 | 2.7–5.4 | 5968 | 5.6 | 5.4–5.8 | 1.5–6.7 |
| 152 | Small intestine | 3 | 0.2 | 0.0–0.6 | 279 | 0.3 | 0.2–0.4 | 0.1–0.3 |
| 153 | Colon | 49 | 4.2 | 2.9–5.5 | 14 188 | 14.9 | 14.5–15.2 | 1.0–2.6 |
| 154 | Rectum | 33 | 3.0 | 1.8–4.1 | 6876 | 7.7 | 7.4–7.9 | 1.9–2.7 |
| 155 | Liver | 24 | 2.4 | 1.3–3.4 | 763 | 0.9 | 0.8–1.0 | 0.8–1.9 |
| 156 | Gall bladder | 39 | 3.7 | 2.4–4.9 | 1074 | 1.1 | 1.0–1.3 | 0.5–1.7 |
| 157 | Pancreas | 36 | 3.7 | 2.4–5.0 | 4958 | 5.1 | 4.9–5.3 | 0.2–1.5 |
| 158 | Peritoneum | 4 | 0.2 | 0.0–0.4 | 184 | 0.3 | 0.2–0.4 | 0.3–0.5 |
| 160 | Nasal cavities | 2 | 0.2 | 0.0–0.5 | 253 | 0.3 | 0.2–0.4 | 0.4–0.9 |
| 161 | Larynx | 8 | 0.8 | 0.2–1.3 | 542 | 0.7 | 0.6–0.9 | 0.6–1.6 |
| 162 | Trachea/bronchus/lung | 68 | 6.9 | 5.2–8.7 | 17 835 | 21.6 | 21.1–22.0 | 1.4–3.0 |
| 163 | Pleura | 2 | 0.2 | 0.0–0.5 | 309 | 0.4 | 0.3–0.5 | 0.1–0.6 |
| 164 | Thymus/heart/mediastinum | 3 | 0.2 | 0.0–0.5 | 108 | 0.2 | 0.1–0.3 | 0.0–0.1 |
| 170 | Bone | 11 | 0.6 | 0.2–1.1 | 281 | 0.6 | 0.5–0.8 | 0.6–1.0 |
| 171 | Connective/soft tissue | 26 | 1.6 | 0.9–2.3 | 770 | 1.3 | 1.1–1.4 | 0.7–1.2 |
| 172 | Melanoma of skin | 8 | 0.6 | 0.1–1.1 | 3171 | 5.4 | 5.1–5.6 | 0.2–0.4 |
| 173 | Non-melanoma skin | 29 | 2.5 | 1.5–3.5 | 14 879 | 17.7 | 17.3–18.0 | 1.1–1.7 |
| 174 | Female breast | 631 | 46.6 | 42.7–50.5 | 46 108 | 72.9 | 72.1–73.7 | 18.2–24.6 |
| 179 | Uterus, part unspecified | 10 | 0.8 | 0.2–1.3 | 455 | 0.7 | 0.5–0.8 | 0.4–1.3 |
| 180 | Cervix | 107 | 8.0 | 6.3–9.6 | 5548 | 10.2 | 9.9–10.5 | 19.3–47.2 |
| 181 | Placenta | 1 | <0.1 | 0.0–0.2 | 7 | <0.1 | 0.0–0.1 | 0.2–0.3 |
| 182 | Body of uterus | 76 | 6.6 | 5.0–8.2 | 5855 | 8.4 | 8.1–8.7 | 1.6–2.3 |
| 183 | Ovary | 104 | 8.0 | 6.3–9.7 | 8061 | 12.2 | 11.9–12.6 | 4.0–6.5 |
| 184 | Other female genital | 11 | 1.2 | 0.4–1.9 | 1570 | 1.7 | 1.5–1.8 | 1.3–2.2 |
| 188 | Bladder | 22 | 1.9 | 1.0–2.7 | 4936 | 5.2 | 5.0–5.4 | 0.5–0.9 |
| 189 | Kidney | 29 | 2.6 | 1.5–3.7 | 2420 | 3.3 | 3.1–3.5 | 0.6–0.9 |
| 190 | Eye | 1 | 0.1 | 0.0–0.3 | 351 | 0.6 | 0.5–0.7 | 0.1–0.4 |
| 191–2 | Brain/nervous system | 46 | 3.0 | 2.0–3.9 | 2161 | 4.2 | 4.0–4.5 | 0.7–1.7 |
| 193 | Thyroid gland | 42 | 3.0 | 1.9–4.0 | 980 | 1.6 | 1.5–1.8 | 1.3–2.8 |
| 194 | Other endocrine glands | 2 | 0.1 | 0.0–0.3 | 247 | 0.5 | 0.4–0.7 | 0.0–0.1 |
| 200–3 | Lymphoma | 107 | 8.9 | 7.0–10.8 | 7077 | 9.8 | 9.5–10.1 | 2.3–3.8 |
| 201 | Hodgkin's disease | 23 | 1.4 | 0.7–2.0 | 724 | 1.6 | 1.4–1.8 | 0.5–0.7 |
| 200/2 | Non-Hodgkin's lymphoma | 64 | 5.6 | 4.1–7.1 | 4470 | 6.1 | 5.8–6.4 | 1.2–2.3 |
| 203 | Multiple myeloma | 20 | 1.9 | 1.0–2.9 | 1883 | 2.1 | 1.9–2.3 | 0.4–0.9 |
| 204–8 | Leukaemia | 84 | 5.6 | 4.3–7.0 | 3188 | 4.6 | 4.3–4.8 | 1.9–2.8 |
| 204 | Lymphoid leukaemia | 32 | 2.1 | 1.3–2.9 | 1308 | 2.0 | 1.8–2.2 | 0.5–1.0 |
| 205 | Myeloid leukaemia | 50 | 3.4 | 2.3–4.5 | 1683 | 2.3 | 2.2–2.5 | 1.0–1.5 |
| 206–8 | Other leukaemia | 2 | 0.2 | 0.0–0.4 | 197 | 0.2 | 0.1–0.3 | 0.3–0.4 |
| | Primary site uncertain[b] | 106 | 10.2 | 8.1–12.3 | 11 130 | 11.5 | 11.2–11.8 | 3.6–14.7 |
| | All sites, excluding 173 | 1858 | 149.9 | 142.6–157.3 | 16 2766 | 219.6 | 218.3–220.9 | 108.9–127.2 |

*n* represents number of cases. [a]Indian rates are based on 1983–87 data. [b]Primary site uncertain includes the following ICD9 codes: 159,165,195–199.

Gandhi, 1993; Dimpy, 1994; Gandhi and Husain, 1994; Kanath, 1996). Classification of the small proportion of names considered to be mixed, for example a non-south Asian forename in combination with a south Asian surname, was undertaken on an individual basis after consultation with panel members. Approximately 80% of the names identified as mixed were female, with a non-south Asian forename in conjunction with a south Asian surname being the most common combination. Where this combination was observed together with a maiden name, the name was classified on the basis of the maiden name. Thus, those with a south Asian maiden name were generally classified as south Asian, and those with a non-south Asian maiden name were classified as non-south Asian. The majority of mixed names with a Christian forename and Moslem surname were classified as non-south Asian because this was considered to be an unlikely combination in a person of true south Asian Moslem ethnic origin.

The denominator for the incidence estimates was derived from the 1991 census, utilizing data available at regional health authority (RHA) level. All those who identified their ethnic

© Cancer Research Campaign 1999

**Table 3** Cancer sites[a] ranked by percentage of total south Asian cases (males)

| South Asian rank | Site | Percentage of cases South Asian | Percentage of cases Non-South Asian | Non-south Asian rank |
|---|---|---|---|---|
| 1 | Lung | 17.8 | 24.0 | 1 |
| 2 | Lymphoma | 12.7 | 5.2 | 6 |
| 3 | Prostate | 8.2 | 13.5 | 2 |
| 4 | Colorectal | 7.9 | 13.3 | 3 |
| 5 | Leukaemia | 5.5 | 2.5 | 9 |
| 6 | Bladder | 5.3 | 7.9 | 4 |
| 7 | Lip, oral cavity, pharynx | 5.1 | 1.9 | 11 |
| 8 | Brain/nervous system | 4.9 | 1.9 | 12 |
| 9 | Liver | 4.4 | 0.8 | 16 |
| 10 | Stomach | 3.6 | 6.0 | 5 |

[a]ICD9: 140–208, excluding 173.

**Table 4** Cancer sites[a] ranked by percentage of total south Asian cases (females)

| South Asian rank | Site | Percentage of cases South Asian | Percentage of cases Non-South Asian | Non-south Asian rank |
|---|---|---|---|---|
| 1 | Breast | 34.0 | 28.3 | 1 |
| 2 | Cervix | 5.8 | 3.4 | 8 |
| 2= | Lymphoma | 5.8 | 4.4 | 5 |
| 4 | Ovary | 5.6 | 5.0 | 4 |
| 5 | Leukaemia | 4.5 | 2.0 | 12 |
| 6 | Colorectal | 4.4 | 12.9 | 2 |
| 7 | Lip, oral cavity, pharynx | 4.1 | 1.1 | 16 |
| 7= | Body of uterus | 4.1 | 3.6 | 7 |
| 9 | Lung | 3.7 | 11.0 | 3 |
| 10 | Oesophagus | 2.5 | 2.1 | 11 |

[a]ICD9: 140–208, excluding 173.

**Table 5** Age-specific rates per 100 000 person–years (1990–92) for the ten most common English south Asian sites[a] (males)

| Site | Ethnic group | 0–14 *n* | 0–14 Rate | 15–29 *n* | 15–29 Rate | 30–49 *n* | 30–49 Rate | 50–74 *n* | 50–74 Rate | 75+ *n* | 75+ Rate |
|---|---|---|---|---|---|---|---|---|---|---|---|
| Lung | South Asian | 0 | 0.0 | 0 | 0.0 | 26 | 5.2 | 272 | 101.4 | 46 | 315.9 |
| | Non-south Asian | 1 | <0.1 | 21 | 0.2 | 1135 | 10.4 | 23 567 | 256.2 | 13 525 | 697.0 |
| Lymphoma | South Asian | 23 | 3.7 | 31 | 6.2 | 58 | 11.6 | 119 | 44.4 | 15 | 103.0 |
| | Non-south Asian | 128 | 1.7 | 571 | 6.1 | 1368 | 12.5 | 4159 | 45.2 | 2109 | 108.7 |
| Prostate | South Asian | 0 | 0.0 | 0 | 0.0 | 1 | 0.2 | 105 | 39.1 | 53 | 364.0 |
| | Non-south Asian | 2 | <0.1 | 3 | <0.1 | 70 | 0.6 | 9837 | 106.9 | 11 535 | 594.4 |
| Colorectal | South Asian | 0 | 0.0 | 7 | 1.4 | 27 | 5.4 | 103 | 38.4 | 16 | 109.9 |
| | Non-south Asian | 1 | <0.1 | 38 | 0.4 | 1065 | 9.7 | 12 313 | 133.8 | 7812 | 402.6 |
| Leukaemia | South Asian | 36 | 5.8 | 11 | 2.2 | 16 | 3.2 | 38 | 14.2 | 5 | 34.3 |
| | Non-south Asian | 326 | 4.4 | 200 | 2.1 | 378 | 3.5 | 1792 | 19.5 | 1349 | 69.5 |
| Bladder | South Asian | 0 | 0.0 | 3 | 0.6 | 14 | 2.8 | 69 | 25.7 | 16 | 109.9 |
| | Non-south Asian | 2 | <0.1 | 18 | 0.2 | 500 | 4.6 | 7195 | 78.2 | 4899 | 252.5 |
| Lip/oral cavity/ | South Asian | 1 | 0.2 | 3 | 0.6 | 25 | 5.0 | 56 | 20.9 | 13 | 89.3 |
| pharynx | Non-south Asian | 12 | 0.2 | 46 | 0.5 | 425 | 3.9 | 1903 | 20.7 | 612 | 31.5 |
| Brain/nervous | South Asian | 19 | 3.1 | 11 | 2.2 | 30 | 6.0 | 35 | 13.1 | 0 | 0.0 |
| system | Non-south Asian | 178 | 2.4 | 227 | 2.4 | 599 | 5.5 | 1698 | 18.5 | 279 | 14.4 |
| Liver | South Asian | 2 | 0.3 | 3 | 0.6 | 9 | 1.8 | 64 | 23.9 | 7 | 48.1 |
| | Non-south Asian | 12 | 0.2 | 14 | 0.2 | 87 | 0.8 | 760 | 8.3 | 346 | 17.8 |
| Stomach | South Asian | 0 | 0.0 | 4 | 0.8 | 18 | 3.6 | 39 | 14.5 | 9 | 61.8 |
| | Non-south Asian | 1 | <0.1 | 18 | 0.2 | 332 | 3.0 | 5424 | 59.0 | 3847 | 198.2 |
| All sites | South Asian | 114 | 18.4 | 111 | 22.3 | 296 | 59.0 | 1182 | 440.6 | 229 | 1572.7 |
| excluding 173 | Non-south Asian | 944 | 12.7 | 2332 | 25.0 | 10 022 | 91.6 | 88 777 | 965.0 | 57 218 | 2948.5 |

[a]ICD9: 140–208, excluding 173.

origin as Indian, Pakistani and Bangladeshi as well as a proportion of a fourth census grouping (other groups Asian, namely East African Asian and Indian subcontinent) were classified as south Asian. To allow for census undercoverage, each RHA was partitioned into its constituent local authority areas (OPCS, 1993*b*) and an appropriate adjustment factor for estimated undercoverage by area type, sex and age group was applied (OPCS, 1994).

Age-specific rates and rates directly standardized to the world standard population with 95% confidence intervals were calculated for malignant neoplasms. Age-standardized rates provide a standard method for presenting cancer rates and allow comparison of results with published data. For each cancer site, the null hypothesis of equal incidence rates for south Asians and non-south Asians was tested against the alternative hypothesis of proportional age-specific rates using a Mantel–Haenszel test (Esteve et

 *© Cancer Research Campaign 1999*

**Table 6** Age-specific rates per 100 000 person–years (1990–92) for the ten most common English south Asian sites[a] (females)

| | | Age group | | | | | | | | | |
|---|---|---|---|---|---|---|---|---|---|---|---|
| | | 0–14 | | 15–29 | | 30–49 | | 50–74 | | 75+ | |
| Site | Ethnic group | *n* | Rate | *n* | Rate | *n* | Rate | *n* | Rate | *n* | Rate |
| Breast | South Asian | 0 | 0.0 | 19 | 3.9 | 262 | 51.5 | 333 | 159.9 | 17 | 101.3 |
| | Non-south Asian | 2 | <0.1 | 271 | 3.0 | 8913 | 82.4 | 25 925 | 256.2 | 10 997 | 289.7 |
| Cervix | South Asian | 0 | 0.0 | 3 | 0.6 | 45 | 8.9 | 56 | 26.9 | 3 | 17.9 |
| | Non-south Asian | 0 | 0.0 | 412 | 4.6 | 2359 | 21.8 | 2042 | 20.2 | 735 | 19.4 |
| Lymphoma | South Asian | 4 | 0.7 | 20 | 4.1 | 20 | 3.9 | 55 | 26.4 | 8 | 47.7 |
| | Non-south Asian | 57 | 0.8 | 409 | 4.6 | 783 | 7.2 | 3279 | 32.4 | 2549 | 67.1 |
| Ovary | South Asian | 3 | 0.5 | 5 | 1.0 | 31 | 6.1 | 58 | 27.9 | 7 | 41.7 |
| | Non-south Asian | 12 | 0.2 | 155 | 1.7 | 1129 | 10.4 | 4767 | 47.1 | 1998 | 52.6 |
| Leukaemia | South Asian | 28 | 4.8 | 12 | 2.5 | 16 | 3.2 | 23 | 11.1 | 5 | 29.8 |
| | Non-south Asian | 236 | 3.4 | 142 | 1.6 | 264 | 2.4 | 1145 | 11.3 | 1401 | 36.9 |
| Colorectal | South Asian | 0 | 0.0 | 1 | 0.2 | 23 | 4.6 | 49 | 23.5 | 9 | 53.6 |
| | Non-south Asian | 0 | 0.0 | 30 | 0.3 | 951 | 8.8 | 9484 | 93.7 | 10 599 | 279.2 |
| Lip/oral cavity/ | South Asian | 1 | 0.2 | 2 | 0.4 | 27 | 5.3 | 40 | 19.2 | 6 | 35.7 |
| pharynx | Non-south Asian | 14 | 0.2 | 34 | 0.4 | 197 | 1.8 | 935 | 9.2 | 591 | 15.6 |
| Body of uterus | South Asian | 0 | 0.0 | 2 | 0.4 | 17 | 3.3 | 53 | 25.5 | 4 | 23.8 |
| | Non-south Asian | 0 | 0.0 | 18 | 0.2 | 444 | 4.1 | 3765 | 37.2 | 1628 | 42.9 |
| Lung | South Asian | 0 | 0.0 | 0 | 0.0 | 7 | 1.4 | 49 | 23.5 | 12 | 71.5 |
| | Non-south Asian | 0 | 0.0 | 11 | 0.1 | 701 | 6.5 | 10 632 | 105.1 | 6491 | 171.0 |
| Oesophagus | South Asian | 0 | 0.0 | 2 | 0.4 | 2 | 0.4 | 34 | 16.3 | 9 | 53.6 |
| | Non-south Asian | 0 | 0.0 | 3 | <0.1 | 82 | 0.8 | 1416 | 14.0 | 1841 | 48.5 |
| All sites | South Asian | 66 | 11.2 | 104 | 21.2 | 543 | 106.8 | 996 | 478.4 | 149 | 887.7 |
| excluding 173 | Non-south Asian | 727 | 10.3 | 2339 | 26.3 | 19 398 | 179.3 | 82 046 | 810.8 | 58 256 | 1534.5 |

[a]ICD9: 140–208, excluding 173.

al, 1994). In effect, the Mantel–Haenzsel test represents a comparison of two age-specific rate curves. For comparison, incidence rates from four cancer registries in India (Ahmedabad, Bombay, Bangalore, Madras) were obtained from published sources (Parkin et al, 1992).

### RESULTS

The Nam Pehchan computer software package identified 5506 south Asian names from the 356 555 names considered in total. After visual inspection, 3482 program positives were confirmed as south Asian and 363 program negatives were also classified as south Asian, yielding a total of 3845 south Asian names in the final classification (1.1%). Using the final classification as reference, Nam Pehchan achieved an overall sensitivity, specificity and positive predictive value of 90.5%, 99.4% and 63.2% respectively.

Age-standardized rates for the major cancer sites are presented in Tables 1 and 2, together with the range of rates for four Indian cancer registries.

### Comparison of south Asian and non-south Asian rates (England)

In males, incidence rates were significantly lower in south Asians compared with non-south Asians for all sites combined and for cancer of the oesophagus, stomach, colon, rectum, pancreas, lung, melanoma of the skin, prostate, testis, bladder and kidney ($P < 0.001$), and for cancer of the pleura ($P < 0.01$). Rates were significantly higher in south Asian males for cancer of the hypopharynx and liver ($P < 0.001$), and for cancer of the gall bladder and Hodgkin's disease ($P < 0.01$).

In females, incidence rates were significantly lower in south Asians compared with non-south Asians for all sites combined and for cancer of the colon, rectum, lung, melanoma of the skin, breast, cervix, ovary and bladder ($P < 0.001$), and for cancer of the stomach, body of uterus, eye, brain/nervous system and other endocrine glands ($P < 0.05$). Rates were significantly higher in south Asian females for cancer of the tongue, mouth, liver and gall bladder ($P < 0.001$), for cancer of the thyroid gland and myeloid leukaemia ($P < 0.01$) and for cancer of the hypopharynx and oesophagus ($P < 0.05$).

A summary of the numerically most important cancer sites is provided in Tables 3 and 4. Differences between south Asians and non-south Asians included the greater relative importance of breast cancer in south Asian females and the higher rank of lymphoma for south Asians of both sexes. Age-specific rates for the most common south Asian sites and corresponding non-south Asian rates are presented in Tables 5 and 6.

The observed and expected number of south Asian cases for each cancer site are presented in Table 7, together with the ratio of observed to expected cases expressed as a percentage. The expected number of south Asian cases represent the total number of cases that would have been observed if the age-specific rates for the south Asian population had been equivalent to the age-specific rates for the non-south Asian population. For example, the number of south Asian male liver cancer cases (85) was more than three times the expected number (26.9).

© Cancer Research Campaign 1999 

**Table 7** Observed and expected number of south Asian cases by cancer site

| | | South Asian males | | | South Asian females | | |
|---|---|---|---|---|---|---|---|
| **ICD9** | **Site** | **Obs.** | **Exp.** | **% O/E** | **Obs.** | **Exp.** | **% O/E** |
| 140 | Lip | 0 | 2.7 | 0 | 0 | 0.7 | 0 |
| 141 | Tongue | 23 | 16.1 | 143 | 25^^^ | 6.9 | 360 |
| 142 | Salivary glands | 9 | 6.5 | 139 | 6 | 5.0 | 119 |
| 143–5 | Mouth | 26 | 21.1 | 123 | 29^^^ | 8.3 | 349 |
| 146 | Oropharynx | 5 | 10.5 | 48 | 5 | 3.0 | 168 |
| 147 | Nasopharynx | 9 | 6.6 | 136 | 2 | 2.7 | 73 |
| 148 | Hypopharynx | 21^^^ | 7.4 | 283 | 7^ | 3.1 | 226 |
| 149 | Other mouth/pharynx | 5 | 3.7 | 136 | 2 | 1.1 | 177 |
| 150 | Oesophagus | 39*** | 88.9 | 44 | 47^ | 32.9 | 143 |
| 151 | Stomach | 70*** | 156.8 | 45 | 40* | 55.2 | 72 |
| 152 | Small intestine | 4 | 6.9 | 58 | 3 | 3.9 | 76 |
| 153 | Colon | 88*** | 208.7 | 42 | 49*** | 148.7 | 33 |
| 154 | Rectum | 65*** | 165.5 | 39 | 33*** | 79.1 | 42 |
| 155 | Liver | 85^^^ | 26.9 | 316 | 24^^^ | 10.6 | 226 |
| 156 | Gall bladder | 26^^ | 14.7 | 177 | 39^^^ | 12.0 | 326 |
| 157 | Pancreas | 47*** | 83.5 | 56 | 36 | 49.5 | 73 |
| 158 | Peritoneum | 9 | 5.3 | 169 | 4 | 3.4 | 118 |
| 160 | Nasal cavities | 6 | 7.9 | 76 | 2 | 3.7 | 54 |
| 161 | Larynx | 48 | 49.9 | 96 | 8 | 8.0 | 100 |
| 162 | Trachea/bronchus/lung | 344*** | 637.7 | 54 | 68*** | 213.2 | 32 |
| 163 | Pleura | 10** | 25.6 | 39 | 2 | 4.5 | 45 |
| 164 | Thymus/heart/mediastinum | 4 | 4.9 | 82 | 3 | 2.4 | 127 |
| 170 | Bone | 21 | 14.8 | 142 | 11 | 11.4 | 96 |
| 171 | Connective/soft tissue | 33 | 27.9 | 118 | 26 | 19.6 | 133 |
| 172 | Melanoma of skin | 7*** | 60.9 | 12 | 8*** | 81.2 | 10 |
| 173 | Non-melanoma skin | 26*** | 293.5 | 9 | 29*** | 201.3 | 14 |
| 174 | Female breast | | | | 631*** | 945.4 | 67 |
| 175 | Male breast | 3 | 6.0 | | 50 | | |
| 179 | Uterus, part unspecified | | | | 10 | 8.7 | 115 |
| 180 | Cervix | | | | 107*** | 171.8 | 62 |
| 181 | Placenta | | | | 1 | 0.4 | 286 |
| 182 | Body of uterus | | | | 76* | 96.0 | 79 |
| 183 | Ovary | | | | 104*** | 151.3 | 69 |
| 184 | Other female genital | | | | 11 | 18.4 | 60 |
| 185 | Prostate | 159*** | 268.8 | 59 | | | |
| 186 | Testis | 28*** | 88.4 | 32 | | | |
| 187 | Penis, other male genital | 5 | 10.8 | 47 | | | |
| 188 | Bladder | 102*** | 211.0 | 48 | 22*** | 51.9 | 42 |
| 189 | Kidney | 49*** | 87.8 | 56 | 29 | 37.7 | 77 |
| 190 | Eye | 9 | 10.4 | 86 | 1* | 8.2 | 12 |
| 191–2 | Brain/nervous system | 95 | 99.8 | 95 | 46* | 63.8 | 72 |
| 193 | Thyroid gland | 13 | 10.0 | 130 | 42^^ | 26.4 | 159 |
| 194 | Other endocrine gland | 10 | 11.4 | 88 | 2* | 8.3 | 24 |
| 200–3 | Lymphoma | 246 | 218.6 | 113 | 107 | 127.0 | 84 |
| 201 | Hodgkin’s disease | 64^^ | 45.6 | 140 | 23 | 29.4 | 78 |
| 200/2 | Non-Hodgkin’s lymphoma | 143 | 136.5 | 105 | 64 | 76.1 | 84 |
| 203 | Multiple myeloma | 39 | 36.5 | 107 | 20 | 21.4 | 93 |
| 204–8 | Leukaemia | 106 | 104.9 | 101 | 84^ | 63.9 | 131 |
| 204 | Lymphoid leukaemia | 55 | 54.2 | 102 | 32 | 29.0 | 110 |
| 205 | Myeloid leukaemia | 50 | 46.0 | 109 | 50^^ | 32.4 | 155 |
| 206–8 | Other leukaemia | 1 | 4.7 | 21 | 2 | 2.5 | 80 |
| | Primary site uncertain[b] | 103*** | 178.9 | 58 | 106 | 117.8 | 90 |
| | All sites, excluding 173 | 1932*** | 2967.8 | 65 | 1858*** | 2667.2 | 70 |

^/*indicate higher/lower age-specific rates in south Asians compared with non-south Asians (see patients and methods) at the following levels of statistical significance: ^/* (*P* <0.05), ^^/** (*P* < 0.01), ^^^/*** (*P* <0.001). %O/E is the ratio of observed to expected number of cases. [b]includes ICD9 codes 159,165,195–199.

 *© Cancer Research Campaign 1999*

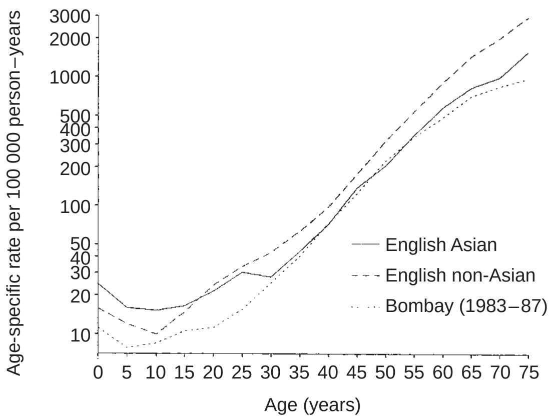

**Figure 1** Age-specific incidence rates (males) per 100 000 person–years (1990–92) for all sites (ICD9: 140–208), excluding non-melanoma skin cancer (ICD9: 173)

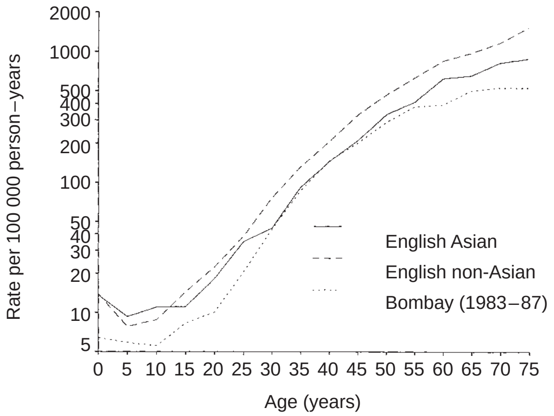

**Figure 2** Age-specific incidence rates (females) per 100 000 person–years (1990–92) for all sites (ICD9: 140–208), excluding non-melanoma skin cancer (ICD9: 173)

Incidence rates have been derived for a large number of cancer sites and, as a consequence, some of the findings deemed to be statistically significant may have occurred merely by chance. The results as a whole are, however, consistent with the hypothesis that rates in migrant populations are intermediate between rates in the country of ethnic origin and those in the country of residence. Further, raised English Asian rates for Hodgkin's disease in males, cancer of the oral cavity and leukaemia in females, and for liver and gallbladder cancer in both sexes confirm the findings of earlier studies on cancer mortality in the migrant south Asian population (Swerdlow et al, 1995; Balarajan et al, 1997).

### Comparison of rates in England with rates for the Indian subcontinent

For the majority of sites, standardized rates for south Asians resident in England (English south Asian) were higher than rates for south Asians resident in India (Indian Asian), but lower than rates for non-south Asians. For testicular cancer in males, lung and ovarian cancer in females, and colorectal and bladder cancer in both sexes, English south Asian rates were closer to, but higher than, Indian Asian rates. For lung, prostate and kidney cancer in males, breast and brain/nervous system cancer in females, and pancreatic cancer in both sexes, English south Asian rates were approximately midway between Indian Asian and non-south Asian rates. For cancer of the brain/nervous system and leukaemia in males and cancer of the uterus, kidney and lymphoma in females, English south Asian rates were closer to, but lower than, non-south Asian rates. For lymphoma in males and leukaemia in females, English south Asian rates were higher than both Indian Asian and non-south Asian rates.

Age-specific rates for English south Asian, non-south Asian and Bombay males and females (all sites combined) are presented in Figures 1 and 2. Non-south Asian rates were higher than Bombay rates for both sexes over all age ranges. In males, English south Asian rates were higher than non-south Asian rates in those under 20, below but closer to non-south Asian rates for ages 20–29, and closer to Bombay rates for adults aged 30 and over. In females, English south Asian rates were as high or higher than non-south Asian rates in those under 14, below but closer to non-south Asian rates for ages 15–29, closer to Bombay rates for adults aged 30–59 and midway between Bombay rates and non-south Asian rates for those aged 60 and over.

### DISCUSSION

In the absence of information on self-ascribed ethnicity, any method of classification of ethnic group is subject to error. In addition, without information on period of residence and birthplace, groups identified as south Asian will be heterogeneous, and include important within-group differences regarding lifestyle and diet, etc. (Senior and Bhopal, 1994). Nonetheless, we believed it sufficiently important to study disease patterns in this group as a whole to warrant the development of a robust methodology for the identification of south Asian names. The pilot study undertaken as a validation exercise suggested acceptable sensitivity and specificity levels for the Nam Pehchan computer software package alone. By combining Nam Pehchan with a range of additional inspection methods, further improvements in accuracy are clearly achievable. The methods outlined could be applied in other situations in which named data are available. Some misclassification of names is likely to have occurred and different methods were used to identify south Asians in the numerator and denominator, but, unless this has led to a disproportionate underestimation or overestimation in either, the rates presented will be sufficiently unbiased.

With the final classification of names as reference, the sensitivity (90.5%) and specificity (99.4%) achieved by Nam Pehchan were very close to the values obtained in the pilot study (91.0% and 99.0% respectively) in which ethnicity had been identified by a specialist tumour registry. The lower positive predictive value (63.2%) compared with the pilot study (87.5%) was due, in part, to the higher proportion of names classified as south Asian by Nam

© Cancer Research Campaign 1999

Pehchan which were considered to be of non-south Asian ethnic origin. The distinction between Moslem names of south Asian and non-south Asian ethnic origin is an area of potential difficulty. Census data indicated that the vast majority of Moslem names under consideration in the current study would be of south Asian ethnic origin. Further, a substantial proportion of the Moslem names classified as non-south Asian were readily identifiable as being of Arab or Turkish rather than south Asian ethnic origin. Nevertheless, it is possible that a small number of Moslem names common to both south Asian and non-south Asian populations will have been misclassified. Names considered to be of Sri Lankan origin were not included in the south Asian classification as Nam Pehchan was unable to accurately classify names of Sinhalese origin. Tamil names are common to both Sri Lanka and south India and it is, therefore, possible that a few names of Sri Lankan origin will have been included in the south Asian classification.

This study provides the most accurate and precise estimates of cancer incidence in the English south Asian population to date. Overall, standardized rates for English south Asians were significantly lower than non-south Asian rates. The higher rates for English south Asians compared with Indian Asians for a number of common sites, including the colon, lung, female breast, lymphoma and leukaemia, suggests that substantial detrimental modifications in exposure and lifestyle have occurred in the migrant south Asian population.

The burden of malignant disease in the English south Asian population currently shows considerable differences from the burden of disease in the non-south Asian population (Tables 3 and 4). These differences, in part, result from the younger age structure of the English south Asian population. Approximately 50% of the English south Asian study population were aged under 25 compared with approximately 33% of the non-south Asian population. Nevertheless, consideration of age-specific rates (Tables 5 and 6) suggests that differences in underlying incidence also exist. For example, the greater relative importance of lymphoma in south Asian males is due to its greater relative importance across all age groups and not just higher rates in those aged 0–14.

Age-standardized rates are presented (Tables 1 and 2) to allow comparisons between English south Asian, non-south Asian and Indian Asian rates. As a weighted sum of age-specific rates, standardized rates may conceal more complex underlying age-specific patterns. Nevertheless, the rates presented serve as a useful summary of cancer incidence in both the English south Asian and non-south Asian study populations.

A comparison of these results with those from an earlier study on cancer mortality in migrants to England and Wales who were born in the Indian subcontinent (Swerdlow et al, 1995) shows significantly raised south Asian rates, in both studies, for Hodgkin's disease in males, cancer of the oral cavity, oesophagus and leukaemia in females, and for cancer of the pharynx (except nasopharynx), liver and gall bladder in both sexes. Rates for thyroid cancer were significantly raised in south Asian males in the mortality study and in south Asian females in the current study. Although the mortality study also found raised south Asian rates for cancer of the oesophagus and pancreas in males, and cervical cancer in females, the current study found significantly lower south Asian rates at these sites. The absence of population denominators and inclusion of only those south Asians born in the Indian subcontinent in the mortality study might explain some of the main differences between the results of the two studies. In addition, differences between incidence and mortality rates may reflect differences in the survival experience of south Asians and non-south Asians.

The comparison of rates between English south Asians and Indian Asians requires caution for the following reasons.

(1) The data on English south Asians cover the period 1990–92 and are based on a population split of approximately 61% Indian, 28% Pakistani and 11% Bangladeshi, whereas the data on Indian Asians cover the period 1983–87 and are based on four regional Indian populations.
(2) No detailed published data were available for Pakistan and Bangladesh.
(3) A substantial proportion of the migration to the United Kingdom from the Indian subcontinent has been from specific regions such as Mirpur in Pakistan and the Punjab in India. The cancer experience in these areas may not be the same as in those covered by the Indian cancer registries.
(4) Further, migrants may not be typical of the resident population in the country of ethnic origin.
(5) There may also be differences in registration practice between registries in the United Kingdom and those in India.
(6) Differences in medical practice between the United Kingdom and the Indian subcontinent will influence the reported incidence of cancer. Incidence rates will reflect the rates at which incidental cancers are discovered by invasive procedures. For example, prostate cancer rates will partly depend on the use of biopsies as a diagnostic tool.
(7) There may also be important differences with regard to access and uptake of health services between the United Kingdom and the Indian subcontinent.

Although the above considerations need to be taken into account, the comparison between Indian Asian and English south Asian rates, nevertheless, serves as a starting point for an assessment of the potential effect of migration on the south Asian population. Bombay cancer registry data were used for the comparison of age-specific rates in Figures 1 and 2 as it is the oldest and largest of the cancer registries in the Indian subcontinent.

The English south Asian population differs from the non-south Asian population both in terms of age distribution and with regard to a range of cultural and socioeconomic factors. There may be ethnic differences with regard to access and uptake of health services that impact on cancer registration. One simple indicator of the quality of cancer registration data is provided by the percentage of cases registered as primary site uncertain. The lower figures for south Asians (5.3% male and 5.7% female) compared with non-south Asians (6.5% male and 6.8% female) suggest that any differences between the two groups relate primarily to age structure rather than differences in the uptake of services and cancer registration practices. Old and frail patients are less likely to be intensively investigated and more likely to be registered as primary site uncertain.

Data from the 1991 census yielded the following approximations for the percentage of English south Asian males (females) born in the United Kingdom: 84% (87%) for ages 0–19, 40% (35%) for ages 20–29, and 3% (4%) for ages 30+. For adults aged 30 and over, their overall cancer risk remained closer to that of the Bombay population. However, English south Asians aged under 30, who represent the first generation to include a substantial proportion born in the United Kingdom, experienced similar or even higher cancer incidence rates than non-south Asians. These

© Cancer Research Campaign 1999

figures suggest that the transition from the lower overall cancer risk of the country of ethnic origin to that of the country of residence is currently more marked in those age groups which include a substantial proportion born in the United Kingdom.

The lower rate for cervical cancer in English south Asians compared with non-south Asians largely resulted from lower rates in the younger age groups (0–49 years), because English south Asian rates in the older age groups (50+ years) were generally higher, suggesting that a cohort effect may be present. A cohort effect may also be present in the English south Asian female breast cancer rates, as younger English south Asian women (20–29 years) had higher age-specific rates than their non-south Asian counterparts (10.1 compared with 6.7 per 100 000 person years for ages 25–29). The upper limit of this age range (29 years) also represented the upper limit of the age groups containing a significant proportion of individuals born in the United Kingdom. This implies that this cohort requires careful monitoring. Low breast and cervical screening rates among English south Asian women (Hoare, 1996) cannot be viewed with complacency.

English south Asian rates for the tongue, mouth and hypopharynx were lower than Indian Asian rates, but higher than non-south Asian rates. This would be consistent with a change in behavioural risk factors, primarily the chewing of tobacco with betel-quid (Tomatis et al, 1990). The greater reduction and lower rates in English south Asian males compared with females is consistent with a report that found that a significantly higher proportion of females compared with males, in at least one of the English south Asian communities (Bangladeshi), included tobacco as a betel-quid chew ingredient (Bedi, 1996).

A number of the findings from this study may be worthy of further investigation. For example:

(1) The lower rate of oesophageal cancer in English south Asian males and higher rate in English south Asian females compared with non-south Asians. These differences may be partly explained by differences between south Asians and non-south Asians and between sexes in the rates of squamous cell carcinoma and adenocarcinoma of the oesophagus.
(2) The higher rates of liver and gall bladder cancer in English south Asians compared with non-south Asians. The higher rate of liver cancer in the English south Asian population may be associated with higher levels of hepatitis B infection.
(3) The rate of lymphoma in English south Asian males is higher than the non-south Asian rate and almost treble the highest Indian Asian rate. The transition from lower Indian Asian rates to higher non-south Asian rates is, at present, more marked for lymphoma than for any other type of cancer. Further, English south Asian male rates are similar to or higher than non-south Asian rates across all age groups (Table 5). This is consistent with an infectious component in the aetiology of lymphoma.

In this study, English south Asians currently exhibit a lower overall cancer risk than non-south Asians. The distribution of age-specific rates for all sites combined, however, gives no cause to assume that this situation will persist. The comparison with Indian registries suggests that exposures associated with common Western cancers have increased. Reductions in the rates of cancer of the oral cavity, pharynx and cervix, although important, do not compensate for these increases. Efforts should be made to modify the adoption of harmful behaviours in this group. It is, therefore, important that English south Asians are targeted in health promotion activities relating to common cancers. The excesses described at certain sites in English south Asians highlight the need for specific preventative interventions within these communities. For sites where differences exist which have not been explained, further studies in this population may provide insight into the aetiology of the conditions.

### ACKNOWLEDGEMENTS

The authors would like to thank panel members for their substantial contribution to the study and acknowledge the active contribution and collaboration of the following cancer registries: Thames Cancer Registry, Trent Cancer Registry, West Midlands Cancer Intelligence Unit, Yorkshire Cancer Registry. This study was supported by a grant from the Medical Research Council. Members of the Cancer Incidence in south Asians Research Group were KK Cheng, C Chilvers, M Coleman, C Cummins, HG Dews, D Forman, J Hiscox, EEM Kernohan, R Maric, P Needham, P Silcocks, C Varghese, H Winter.

### REFERENCES

Balarajan R and Raleigh VS (1997) Patterns of mortality among Bangladeshis in England and Wales. *Ethnic Health* **2**: 5–12
Balarajan R, Bulusu L, Adelstein AM and Shukla V (1984) Patterns of mortality among migrants to England and Wales from the Indian subcontinent. *Br Med J Clin Res Edition* **289**: 1185–1187
Barker RM and Baker MR (1990) Incidence of cancer in Bradford Asians. *J Epidemiol Community Health* **44**: 125–129
Bedi R (1996) Betel-quid and tobacco chewing among the United Kingdom's Bangladeshi community. *Br J Cancer* (suppl. 29) **74**: S73–S77
Bradford Health Authority. Nam Pehchan computer software (Version 1.1). New Mill, Victoria Road, Saltaire, Shipley BD18 3LD, UK. Computer Services, City of Bradford Metropolitan Council (Dept. 13), Britannia House, Bradford BD1 1HX, UK
Dimpy MK (1994) *Sikh Baby Names*. Star Publications: New Delhi
Donaldson LJ and Clayton DG (1984) Occurrence of cancer in Asians and non-Asians. *J Epidemiol Community Health* **38**: 203–207
Est'eve J, Benhamou E and Raymond L (1994) Descriptive epidemiology. In *Statistical Methods in Cancer Research*, vol 4. IARC Scientific Publications: number 128. IARC: Lyon
Gandhi M (1993) *The Penguin Book of Hindu Names*. Penguin Books: New Delhi
Gandhi M and Husain O (1994) *The Complete Book of Muslim and Parsi Names*. Indus: New Delhi
Grulich AE, McCredie M and Coates M (1995) Cancer incidence in Asian migrants to New South Wales, Australia. *Br J Cancer* **71**: 400–408
Hoare T (1996) Breast screening and ethnic minorities. *Br J Cancer* (suppl. 29) **74**: S38–S41
Kanath MV (1996) *Jaico Book of Baby Names* Jaico Publishing House: Bombay
Matheson LM, Dunnigan MG, Hole D and Gillis CR (1985) Incidence of colo-rectal, breast and lung cancer in a Scottish Asian population. *Health Bull Edinburgh* **43**: 245–249
Muir KR, Parkes SE, Mann JR, Stevens MCG and Cameron AH (1992) Childhood cancer in the West Midlands: incidence and survival, 1980–1984, in a multi-ethnic population. *Clin Oncol* **4**: 177–182
OPCS (Office of Population Censuses and Surveys) (1993*a*) *1991 Census Report for England: Regional Health Authorities*. HMSO: London
OPCS (Office of Population Censuses and Surveys) (1993*b*) *1991 Census. County Reports*. HMSO: London
OPCS (Office of Population Censuses and Surveys) (1994) *1991 Census. User Guide 58: Undercoverage in Great Britain*. HMSO: London
Parkin DM, Muir CS, Whelan SK, Gao YT, Ferlay J and Powell J (1992) *Cancer Incidence in Five Continents*, vol 6. IARC Scientific Publications: number 120. IARC: Lyon
Patel V (1992) *Babies' Names from the Indian Sub-Continent*. Foulsham: Bury St. Edmunds
Powell JE, Parkes SE, Cameron AH and Mann JR (1994) Is the risk of cancer increased in Asians living in the UK? *Arch Dis Childhood* **71**: 398–403
Senior PA and Bhopal R (1994) Ethnicity as a variable in epidemiological research. *Br Med J* **309**: 327–330

© Cancer Research Campaign 1999 

Stiller CA, McKinney PA, Bunch KJ, Bailey CC and Lewis IJ (1991) Childhood cancer and ethnic group in Britain: a United Kingdom Children's Cancer Study Group (UKCCSG) study. *Br J Cancer* **64**: 543–548

Swerdlow AJ, Marmot MG, Grulich AE and Head J (1995) Cancer mortality in Indian and British ethnic immigrants from the Indian subcontinent to England and Wales. *Br J Cancer* **72**: 1312–1319

Tomatis L, Aitio A, Day NE, Heseltine E, Kaldor J, Miller AB, Parkin DM and Riboli E (1990) *Cancer: Causes, Occurrence and Control*. IARC Scientific Publications: number 100. IARC: Lyon

Varghese C, Barrett JH, Johnston C, Shires M, Rider L and Forman D (1996) High risk of lymphomas in children of Asian origin: ethnicity or confounding by socioeconomic status? *Br J Cancer* **74**: 1503–1505

*© Cancer Research Campaign 1999*
